# Impact of estradiol, testosterone and their ratio on left and right auricular myofilament function in male and female patients undergoing coronary artery bypass grafting

**DOI:** 10.1186/s12872-023-03582-4

**Published:** 2023-11-04

**Authors:** C. Bening, B. Genser, D. Keller, S. Müller-Altrock, D. Radakovic, K. Penov, M. Hassan, I. Aleksic, R. Leyh, N. Madrahimov

**Affiliations:** 1 Department of Thoracic and Cardiovascular Surgery, University Hospital Wuerzburg Zentrum Operative Medizin, Oberduerrbacherstr. 6, 97080 Wuerzburg, Germany; 2 Medical Faculty Mannheim, Center for Preventive Medicine, Heidelberg University, Digital Health Baden-Württemberg (CPD-BW), Heidelberg , Germany

**Keywords:** Sex differences, E/T ratio, 17ßEstradiol, Testosterone, Skinned fiber

## Abstract

**Background:**

The impact of sex hormones on right and left auricular contractile apparatus function is largely unknown. We evaluated the impact of sex hormones on left and right heart contractility at the level of myocardial filaments harvested from left and right auricles during elective coronary artery bypass surgery.

**Methods:**

150 patients (132 male; 18 female) were enrolled. Preoperative testosterone and estradiol levels were measured with Immunoassay. Calcium induced force measurements were performed with left- and right auricular myofilaments in a skinned fiber model. Correlation analysis was used for comparison of force values and levels of sex hormones and their ratio.

**Results:**

Low testosterone was associated with higher top force values in right-sided myofilaments but not in left-sided myofilaments for both sexes (p = 0.000 in males, p = 0.001 in females). Low estradiol levels were associated with higher top force values in right-sided myofilaments (p 0.000) in females and only borderline significantly associated with higher top force values in males (p 0.056). In females, low estradiol levels correlated with higher top force values in left sided myofilaments (p 0.000). In males, higher Estradiol/Testosterone ratio (E/T ratio) was only associated with higher top force values from right auricular myofilaments (p 0.04) In contrast, in females higher E/T ratio was associated with lower right auricular myofilament top force values (p 0.03) and higher top force values in left-sided myofilaments (p 0.000).

**Conclusions:**

This study shows that patients’ comorbidities influence left and right sided contractility and may blur results concerning influence of sex hormones if not eliminated. A sex hormone dependent influence is obvious with different effects on the left and right ventricle. The E/T ratio and its impact on myofilament top force showed divergent results between genders, and may partially explain gender differences in patients with cardiovascular disease.

## Introduction

Clinical and basic science studies have shown important sex differences in cardiac structure and function [1–5]. Although an impact of sex hormones is frequently discussed, data from basic science studies show conflicting results and cannot simply be extrapolated to clinical settings. Clinical studies mainly focus on functional and morphological differences between genders. Studies on humans evaluating the impact of sex hormones on contractility at the level of myocardial filaments are sparse and limited by small study populations [6–8]. The ambiguity of knowledge in this topic is described by Ventetuolo and Subramanya et al., who stated that estradiol and testosterone have important but controversial and in part unknown roles for left and right auricular function [9–11].

According to the available literature, it seems obvious that sex hormones may have a different impact on right and left auricular function. The MESA study (Multi-Ethnic Study of Atherosclerosis), which evaluated the correlation between serum level of sex hormones and RV and LV function and structure with cardiac MRI [9, 11] showed that women tended to have higher RVEF and lower RVSV, RV mass, RVEDV and RVESV than men [9]. However, concerning higher RV-EF, these data were derived from women with exogenous estradiol intake. Furthermore, higher testosterone levels were associated with greater RV mass and larger RV volumes in men but not in women [9]. The same group demonstrated that higher testosterone levels were associated with a modest increase in left auricular mass and possibly better LV function in both genders [11]. In contrast, high estradiol levels were only associated with increased LV mass in men.

Dai et al. recommended investigating the combination of estrogens and androgens together as a ratio taking into account that the imbalance of hormones might be an important factor for sex specific cardiac function [6]. The importance of the interaction between these sex hormones were underlined by data from the MESA study. This study proved that a high estradiol/testosterone ratio (/E/T ratio) was associated with lower RV volumes and presumably better RV function in men, which was not identified by single sex hormone analysis [9]. However, the impact of sex hormones, their ratio on the level of the contractile apparatus of the left and right ventricle was not evaluated.

To elucidate whether sex hormones and their ratio may influence the contractile apparatus we analyzed the impact of sex hormones and their ratio on the intrinsic functional state of human right and left auricular function by using calcium induced force measurements of skinned human fibers from the right and left auricle from patients with coronary heart disease undergoing elective coronary artery bypass grafting (CABG).

## Methods

### Study sample

The study included one hundred fifty patients undergoing elective on-pump CABG between January 2019 and September 2019. We excluded patients with valve pathologies, reoperations and emergency indications. Since the first regression analysis revealed, that patients with diabetes mellitus, atrial fibrillation and peripheral arterial disease develop significant lower top force values, we also excluded patients presenting these diagnoses and performed a second regression analysis. Metabolic syndrome included arterial hypertension, diabetes mellitus II, dyslipidemia and obesity (BMI > 30 kg/m2). Seventy-one patients remained for the second regression analysis (63 males and 8 females). All patients were informed about the aim of the study and gave their written consent to participate. The University Hospital ethics committee approved the study (IRB approval: 143/17-sc 6.10.2017).We collected clinical findings and preoperative blood samples, taken prior to induction of anesthesia. Blood samples were immediately sent to the laboratory and stored at -80 °C. All data were recorded pseudonymously in a departmental database.

Preoperative clinical chemistry included HbA1C, creatinine, glomerular filtration rate (GFR) and N-terminal pro brain natriuretric peptid (NT-proBNP). The blood samples for the measurement of Estradiol and Testosterone were routinely taken in the morning and at the same time for all patients. Estradiol and testosterone were measured with automated Immunoassay system (Cobas e601).

### Tissue harvest

All patients underwent on pump aortocoronary bypass grafting (CABG). Right auricular tissue was resected for venous cannulation for institution of cardiopulmonary bypass. The left auricle was removed after aortic cross clamping and antegrade infusion of Buckberg blood cardioplegia for the purpose of stroke prevention (4 min).

### Myofilament preparation

Our experimental setup has been described in full detail before [3, 4]. Briefly, the intraoperatively resected tissue was transported in ice-cold oxygenated cardioplegic solution, containing BDM (Sigma Aldrich Chemie GmbH, Steinheim, Germany). For the skinning procedure, the trabeculae were excised and permeabilized with Triton-X solution (Sigma Aldrich Chemie GmbH, Steinheim, Germany). The skinned myofilaments were then attached to a force transducer and a forceps and the experiments were conducted by immersing the myofilaments in twelve bowls with increasing calcium concentrations. The calcium concentration is displayed as logarithmic calcium concentration (pCa), which is a negative decadic logarithm. We started with the lowest calcium concentration at pCa 7.0 and increasing at 6.5, 6.0, 5.75, 5.5, 5.4, 5.3, 5.2, 5.1, 5.0, 4.75, 4.52. Length changes, recorded by the force transducer, were recorded and stored in a database.

### Statistical methods

Exploratory data analysis included descriptive statistics as well as boxplots, histograms and kernel density estimates to visualize the distribution of force values and study variables. The five replicates of force measurements for each patient at a specific calcium concentration tissue sample were aggregated to means for all further statistical analyses. We used three parameters for the assessment of cardiac contractility: top force value, calcium sensitivity (calcium concentration of half maximal activation) and steepness of the curves defined as Hill slope. Data are presented as mean ± standard deviation. Sex hormones were analyzed as high and low sex hormone concentrations according to the mean value. Treshold was 22.8 pg/ml for 17ß-Estradiol and 3.7 ng/ml for testosterone. E/T ratio was grouped in center (Q2, Q3) and tails (Q1, Q4).

We used spline regression models to visualize the functional relationship between force values and the -log10 of the calcium concentrations series (7.0, 6.5, 6.0, 5.75, 5.5, 5.4, 5.3, 5.2, 5.1, 5.0, 4.75, 4.52). Calcium concentrations were transformed by a restricted cubic spline function with 6 knots placed based on Harrell’s recommended percentiles. All force pCa curves were fitted for measurements obtained from the left and right heart muscle separately. In addition to curves for the total population we fitted curves for subgroups to explore the impact of study variables (age, gender, BMI, Estradiol, testosterone, HbA1c, lung function (FEV), renal function (GFR), PAD, atrial fibrillation, diabetes, metabolic syndrome, EuroScore). Atrial fibrillation, diabetes mellitus and PAD significantly decreased force development of right- and left sided myofilaments and were subsequently excluded in a second regression analysis. The second parametric regression analysis excluding patients with atrial fibrillation, diabetes mellitus and PAD included 63 male and 8 female patients.

Curves for Estradiol and Testosterone were additionally fitted stratified by sex to explore whether hormones act differently on force in males and females. Continuous variables were categorized in 4 groups defined by quartiles of the observed distribution. Finally, we used non-linear regression to model the force values as a function of the -log10 of the calcium concentrations. As functional relationship we assumed a sigmoid form reflecting the Hill equation with four parameters [12]. One parameter (bottom force, i.e. the force at pCa = 7.0) was fixed to zero, the other three parameters (top force, pCa50 and Hill slope) were estimated from the data using nonlinear least-squares estimation. Top force quantifies the maximum force reached by the muscle sample stimulated at pCa = 4.52; pCa50 quantifies the calcium sensitivity of the muscle tissue sample as the concentration required for 50% of maximum force, whereas the Hill slope describes the slope of the curve at the midpoint pCa50. In addition, we calculated t-tests to compare the parameter estimates obtained from the different subgroups.

All statistical analyses were conducted using the software STATA (StataCorp. 2017. Stata Statistical Software: Release 15. College Station, TX: StataCorp LLC).

## Results

The study cohort consisted of 150 patients (132 men (88%) and 18 women (12%). Demographic data are depicted in Table 1.

**Table 1:** Patient’s characteristics

Characteristics	TOTAL	Male	Female	P- value
Number of patients n(%)	150 (100%)	132 (88)	18 (12)	
Age (y, mean ± SD)	67.9 ± 9.6	67.4 ± 9.7	71.0 ± 7.7	0.133
Height (cm, mean ± SD)	171.8 ± 7.8	173.4 ± 6.5	159.7 ± 5.4	**= 0.000**
Weight (kg, mean ± SD)	86.7 ± 15.1	88.4 ± 14.7	73.8 ± 5.4	**= 0.000**
Body Mass Index (kg/m², mean ± SD)	29.3 ± 4.1	29.3 ± 4.1	29.9 ± 4.3	0.856
Body Surface Area (m², mean ± SD)	2.0 ± 0.2	2.0 ± 0.2	1.8 ± 0.1	**= 0.000**
EuroSCOREII (mean ± SD)	2.6 ± 12.3	2.7 ± 13.1	2.3 ± 1.7	0.078
Diabetes mellitus (n, %)	54 (36)	48 (36.14)	6 (33)	0.426
1: Type I	1 (0.6)	1 (0.7)	0	
2: Type II without medication	10 (6.6)	7 (5.3)	3 (16.6)	
3: II with oral medication	23 (15.3)	20 (15.2)	3 (16.6)	
4: II with insulin	3 (2)	3 (2.3)	0	
5: II with oral med. + insulin	16(10.6)	16 (12.1)	0	
6: Prediabetes	1 (0.6)	1 (0.7)	0	
Hypertension (n, %)	129 (86)	112 (84.8)	17 (94.4)	0.271
Metabolic Syndrome (n, %)	37 (24.6)	97 (73.5)	14 (77.7)	0.746
Dyslipidemia (n, %)	125 (83.3)	113 (85.6)	12 (66.6)	0.278
Nicotine abuse (n, %)	47 (31.3)	43 (23.6)	4 (22.2)	0.721
ACE inhibitors (n, %)	82 (54.6)	80 (60.6)	2 (11.1)	0.388
Ca channel antagonists (n, %)	37 ((24.6)	35 (26.5)	2 (11.1)	**0.041**
Statines (n, %)	125 (83.3)	113 (85.6)	12 (66.6)	0.233
FEV1% Predicted (mean ± SD)	90.7 ± 19.5	88.7 ± 18.4	106.4 ± 20.4	**0.003**
Peripheral arterial disease (n, %)	19 (12.6)	16 (12.1)	3 (16.6)	0.569
Atrial Fibrillation (n, %)	27 (18)	24 (18.2)	3 (16.6)	0.522
1: Permanent (n, %)	7 (4.6)	7 (5.3)	0	
2: Paroxysmal (n, %)	20 (13.3)	17 (12.9)	3 (16.6)	
Creatinine (mg/dl, mean ± SD)	1.22 ± 0.06	1.25 ± 0.07	1.05 ± 0.09	**0.049**
GFR (MDRD, ml/min/1.73 m², mean ± SD)	71.4 ± 21.8	72.5 ± 21.6	63.6 ± 21.4	0.101
HbA1C (IFCC, mmol/mol)	44.2 ± 11.1	44.5 ± 11.6	41.9 ± 5.47	0.895
Estradiol (pg/ml, mean ± SD))	38.2 ± 170.7	40.2 ± 176.4	9.4 ± 3.9	**= 0.000**
Testosterone (ng/ml, mean ± SD))	3.8 ± 2.2	4.1 ± 2.0	0.5 ± 1.2	**= 0.000**
NTPro-BNP (pg/ml, mean ± SD)	1207.4 ± 3342.5	1267.4 ± 3532.3	741.8 ± 894.8	0.578
Previous (last 12 months) STEMI (n, %)	7 (4.6)	6 (4.5)	1 (5.5)	0.343
Previous (last 12 months NSTEMI	25 (16.6)	24 (18.2)	1 (5.5)	0.158
Previous PCI	28 (18.6)	27 (20.5)	1 (5.5)	0.063
Previous CABG	0	0	0	
3 vessel disease	125 (83.3)	109 (82.6)	16 (88.8)	0.063
2 vessel disease	(25 16.6)	23 (17.4)	2 (11.1)	0.252
1 vessel disease	0	0	0	
Number arterial grafts (mean ± SD)	1.4 ± 0.8	1.4 ± 0.8	1.4 ± 0.8	0.69
Number venous grafts (mean ± SD)	1.75 ± 0.75	1.7 ± 0.8	1.8 ± 0.7	**0.024**
Number of bypasses (mean ± SD	3.22 ± 0.8	3.16 ± 0.8	3.28 ± 0.8	0.208
LVEF (%) ± SD	50 ± 14.6	53 ± 10.3	47 ± 19.0	0.129
TAPSE mm ± SD	22,5 ± 3.5	22 ± 3.9	21 ± 3.0	0.199
sPAP (mmHg ± SD)	29,8 ± 9.7	28.8 ± 9.4	30.8 ± 9.9	0.246
Left ventricular stroke volume (ml ± SD)	82.5 ± 20.6	88.1 ± 21.3	77 ± 19.9	0.064
Ovariectomy (n, %)			0 (0)	
Hysterectomy (n, %)			0 (0)	
Hormone replacement therapy (n, %)			0 (0)	

Both groups were comparable except for lower height (159.7 ± 5.4 cm versus 173.4 ± 6.5 cm, p < 0.00), and body surface area (1.8 ± 0.1 m^2^ versus 2.0 ± 0.2 m^2^, p < 0.00), better FEV1 (106.4 ± 20.4% versus 88.7 ± 18.4%, p = 0.003) and lower serum creatinine in women. Serum concentrations of estradiol and testosterone were significantly lower in women.

**Fig. 1:**
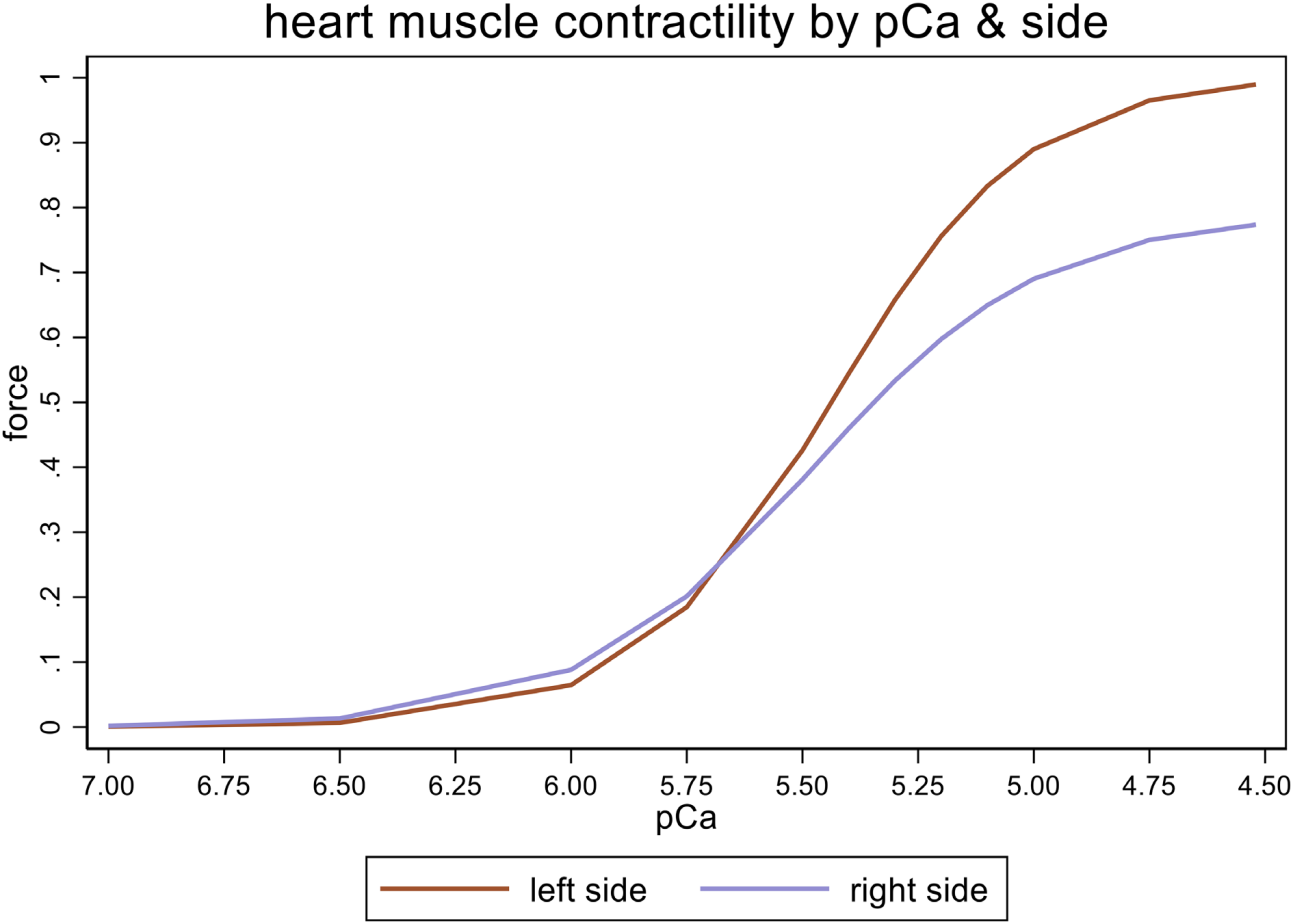
Force values of right heart myofilaments were lower than left side for all patients

We observed higher top force values for left-sided myofilaments (p = 0.000) and decreased calcium sensitivity (pCa_50,_ p = 0.044) in all patients compared to right –sided myofilaments (Fig. 1**)**.

Calcium concentrations (negative decadic logarithm) dependent force development (mN).

However, steepness of the curve (Hill slope) did not differ between left- and right-sided myofilaments (p = 0.05).

We could not identify a significant impact of gender or age on right- or left myofilament contractility (Tables 2 and 3).

**Table 2:** Impact of gender on left and right heart contractility

	T-statistics	P-value	Coefficient
Male vs. female Left Top Force	0.66	0.511	Males = 0.99 Females = 1.02
Male vs. female Left pCa 50	1.07	0.283	Males = 5.43 Females = 5.46
Male vs. female Left Hill slope	0.85	0.397	Males = 2.09 Females = 1.83
Male vs. female Right Top Force	0.75	0.453	Males = 0.79 Females = 0.75
Male vs. female Right pCa 50	0.81	0.419	Males = 5.47 Females = 5.52
Male vs. female Right Hill slope	0.50	0.617	Males = 1.76 Females = 1.60

**Table 3:** Impact of low versus high age on left and right heart contractility

	T-statistics	P-value	Coefficient
Low vs. high age Left Top Force	1.36	0.175	Low = 1.025 High = 0.977
Low vs. high age Left pCa 50	1.44	0.150	Low = 5.419 High = 5.45
Low vs. high age Left Hill Slope	0.69	0.489	Low = 2.15 High = 1.98
Low vs. high age Right Top Force	1.13	0.258	Low = 0.81 High = 0.76
Low vs. high age Right pCa 50	0.83	0.406	Low = 5.46 High = 5.50
Low vs. high age Right Hill slope	0.48	0.633	Low = 1.79 High = 1.69

Based on mean values we analyzed high and low sex hormone concentrations. Threshold was 22.8 pg/ml for 17ß-Estradiol and 3.7 ng/ml for testosterone (Tables 4 and 5).

**Table 4:** Impact of low and high (median as cut off) estradiol hormone levels on left and right heart contractility

All patients (n = 150)	T-statistics	P-value	Coefficient
Estradiol LAA/Male Top force	1.66	0.096	Low value = 0.97 High value = 1.02
Estradiol LAA/Male pCa 50	0.73	0.466	Low value = 5.42 High value = 5.44
Estradiol LAA/Male Hill slope	0.23	0.822	Low value = 2.13 High value = 2.06
Estradiol RAA/Male Top force	2.61	**0.004**	Low value = 0.85 High value = 0.75
Estradiol RAA/Male pCa 50	0.05	0.958	Low value = 5.47 High value = 5.47
Estradiol RAA/Male Hill slope	0.31	0.754	Low value = 1.80 High value = 1.72
Estradiol LAA/Female Top force	1,39	0.163	Low value = 1.12 High value = 0.99
Estradiol LAA/Female pCa 50	0.63	0.528	Low value = 5.44 High value = 5.49
Estradiol LAA/Female Hill slope	0.30	0.762	Low value = 1.91 High value = 1.75
Estradiol RAA/Female Top force	1.94	*0.052*	Low value = 0.87 High value = 0.69
Estradiol RAA/Female pCa 50	0.63	0.528	Low value = 5.52 High value = 5.51
Estradiol RAA/Female Hill slope	0.30	0.761	Low value = 1.48 High value = 1.71

**Table 5:** Impact of low and high (median as cut off) Testosterone hormone levels on left and right heart contractility

All patients (n = 150)	T-statistics	P-value	Coefficient
Testosterone LAA/Male Top force	1.01	0.313	Low value = 1.01 High value = 0.97
Testosterone LAA/Male pCa 50	0.35	0.724	Low value = 5.42 High value = 5.43
Testosterone LAA/Male Hill slope	0.15	0.882	Low value = 2.12 High value = 2.07
Testosterone RAA/Male Top force	4.53	**0.000**	Low value = 0.88 High value = 0.71
Testosterone RAA/Male pCa 50	0.27	0.790	Low value = 5.48 High value = 5.47
Testosterone RAA/Male Hill slope	0.16	0.872	Low value = 1.78 High value = 1.73
Testosterone LAA/Female Top force	0.80	0.424	Low value = 1.01 High value = 0.98
Testosterone LAA/Female pCa 50	0.04	0.970	Low value = 5.43 High value = 5.43
Testosterone LAA/Female Hill slope	0.04	0.968	Low value = 2.05 High value = 2.06
Testosterone RAA/Female Top force	3.96	**0.000**	Low value = 0.86 High value = 0.72
Testosterone RAA/Female pCa 50	0.54	0.588	Low value = 5.49 High value = 5.47
Testosterone RAA/Female Hill slope	0.54	0.965	Low value = 1.74 High value = 1.73

Low 17ß-estradiol levels were associated with higher top force in males’ right-sided myofilaments (p = 0.004). In women, a trend towards low 17ß-estradiol concentrations and higher top force values in right-sided myofilaments was observed (p = 0.052). 17ß-estradiol had no impact on left auricular myofilament top force values in both sexes.

There was a significant correlation of low testosterone serum concentration and higher top force values in right-sided myofilaments in males (p = 0.000) and females (p = 0.000). In contrast, there was no impact of testosterone on left-sided myofilament contractility.

E/T ratio analysis demonstrated (Table 6) that high E/T ratio was associated with higher top force in right-sided myofilaments in men (p = 0.023) but no effect on left-sided myofilaments. The E/T ratio had no effect on women’s right- or left-sided myofilament contractility.

**Table 6:** Sex hormone, E/T ratio and correlation to Top Force values

	T-statistics	P-value	Coefficient
All patients (n = 150) LAA vs. RAA Top Force	8.10	**0.000**	LAA = 1.00 RAA = 0.79
LAA vs. RAA pCa 50	2.02	**0.044**	LAA = 5.43 RAA 5.48
LAA vs. RAA Hill slope	1.90	0.057	LAA = 2.06 RAA = 1.74
E/T ratio and pCa ;Men, LAA, Top Force	0.78	0.44	center = 0.98 tails = 1.01
E/T ratio and pCa ;Men, RAA, Top Force	2.27	**0.023**	Low value = 0.75 High value: 0.83
E/T ratio and pCa ;Women, LAA, Top Force	0.48	0.6	center = 1.00 tails: 1.03
E/T ratio and pCa ;Women, RAA, Top Force	1.20	0.2	Low value = 0.85 High value = 0.71

Analysis of comorbidities (Table 7, **at the end of document**) showed a negative impact of atrial fibrillation on both left- and right-sided myofilaments, of peripheral arterial disease (PAD) on left-sided myofilaments and of diabetes mellitus on right-sided myofilaments.

**Table 7:** Influence of comorbidities on left and right heart contractility

All patients (n = 150)	T-statistics	P-value	Coefficient
Atrial fibrillation LAA Top force	6.27	**0.000**	no = 1.04 yes = 0.80
Atrial fibrillation LAA pCa 50	0.30	0.763	no = 5.43 yes = 5.42
Atrial fibrillation LAA Hill slope	1.11	0.267	no = 2.10 yes = 1.82
Atrial fibrillation RAA Top force	6.27	**0.000**	no = 0.81 yes = 0.66
Atrial fibrillation RAA pCa 50	0.30	0.763	no = 5.47 yes = 5.51
Atrial fibrillation RAA Hill slope	1.11	0.267	no = 1.74 yes = 1.72
PAOD LAA Top force	2.88	**0.004**	no = 1.01 yes = 0.90
PAOD LAA pCa 50	0.60	0.546	no = 5.43 yes = 5.42
PAOD LAA Hill slope	0.08	0.937	no = 2.06 yes = 2.03
PAOD RAA Top force	0.63	0.531	no = 0.79 yes = 0.75
PAOD RAA pCa 50	0.20	0.844	no = 5.48 yes = 5.48
PAOD RAA Hill slope	0.15	0.882	no = 1.73 yes = 1.78
Arterial hypertension LAA Top force	1.61	0.107	no = 0.94 yes = 1.01
Arterial hypertension LAA pCa 50	0.20	0.844	no = 5.42 yes = 5.43
Arterial hypertension LAA Hill slope	1.15	0.882	no = 1.83 yes = 2.09
Arterial hypertension RAA Top force	0.36	0.718	no = 0.80 yes = 0.78
Arterial hypertension RAA pCa 50	0.36	0.722	no = 5.45 yes = 5.48
Arterial hypertension RAA Hill slope	2.10	**0.036**	no = 1.55 yes = 1.77
IDDM LAA Top force	0.72	0.472	no = 1.01 yes = 0.98
IDDM LAA pCa 50	1.86	0.063	no = 5.41 yes = 5.47
IDMM LAA Hill slope	0.63	0.528	no = 2.14 yes = 1.97
IDDM RAA Top force	4.03	**0.000**	no = 0.83 yes = 0.70
IDDM RAA pCa 50	1.29	0.198	no = 5.46 yes = 5.52
IDMM RAA Hill slope	0.53	0.599	no = 1.78 yes = 1.66
Metabolic syndrome LAA Top force	0.43	0.664	no = 1.01 yes = 0.99
Metabolic syndrome LAA pCa 50	0.31	0.759	no = 5.43 yes = 5.43
Metabolic syndrome LAA Hill slope	0.29	0.771	no = 2.13 yes = 2.03
Metabolic syndrome RAA Top force	0.21	0.832	no = 0.78 yes = 0.79
Metabolic syndrome RAA pCa 50	0.87	0.386	no = 5.44 yes = 5.49
Metabolic syndrome RAA Hill slope	0.37	0.710	no = 1.83 yes = 1.71

After exclusion of these confounders a second parametric regression analysis was performed and 71 patients remained (63 males and 8 females). Results are depicted in Table 8 (**at the end of document).**

**Table 8:** Regression analysis after exclusion of patients with atrial fibrillation, peripheral arterial disease and diabetes mellitus II

	T-statistics	P-value	Coefficient
All patients (n = 71) LAA vs. RAA Top Force	7.79	**0.000**	LAA = 1.06 RAA = 0.87
LAA vs. RAA pCa 50	1.88	0.060	LAA = 5.42 RAA 5.45
LAA vs. RAA Hill slope	1.95	0.051	LAA = 2.21 RAA = 1.81
Male vs. female Left Top force	0.48	0.629	Males = 1.06 Females = 1.07
Male vs. female Left pCa 50	1.07	0.283	Males = 5.41 Females = 5.43
Male vs. female Left Hill slope	0.98	0.327	Males = 2.29 Females = 1.72
Male vs. female Right Top force	0.73	0.465	Males = 0.88 Females = 0.79
Male vs. female Right pCa 50	0.81	0.419	Males = 5.45 Females = 5.45
Male vs. female Right Hill slope	0.61	0.541	Males = 1.83 Females = 1.66
Low vs. high Age Left Top Force	0.27	0.786	Low = 1.06 High = 10.6
Low vs. high Age Left pCa 50	1.08	0.281	Low = 5.41 High = 5.43
Low vs. high Age Left Hill Slope	0.57	0.569	Low = 2.22 High = 2.21
Low vs. high Age Right Top Force	0.85	0.396	Low = 0.89 High = 0.83
Low vs. high Age Right Top Force	0.83	0.406	Low = 5.45 High = 5.46
Low vs. high Age Right Top Force	0.52	0.603	Low = 1.85 High = 1.74
Estradiol LAA/Male Top force	1.43	0.180	Low = 1.03 High = 1.09
Estradiol LAA/Male pCa 50	0.73	0.466	Low = 5.41 High = 5.42
Estradiol LAA/Male Hill Slope	0.13	0.893	Low = 2.17 High = 2.41
Estradiol RAA/Male Top force	1.92	**0.056**	Low = 0.92 High = 0.84
Estradiol RAA/Male pCa 50	0.39	0.696	Low = 5.48 High = 5.42
Estradiol RAA/Male Hill Slope	0.04	0.970	Low = 1.73 High = 1.97
Estradiol LAA/Female Top force	5.09	**0.000**	Low = 1.57 High = 0.99
Estradiol LAA/Female pCa 50	0.24	0.811	Low = 5.37 High = 5.45
Estradiol LAA/Female Hill Slope	0.30	0.761	Low = 2.18 High = 1.66
Estradiol RAA/Female Top force	9.22	**0.000**	Low = 1.64 High = 0.67
Estradiol RAA/ Female pCa 50	0.35	0.725	Low = 5.40 High = 5.47
Estradiol RAA/ Female Hill Slope	0.51	0.609	Low = 1.51 High = 1.72
Testosterone LAA/ Male Top Force	0.23	0.820	Low = 1.07 High = 1.05
Testosterone LAA/ Male pCa 50	0.35	0.724	Low = 5.41 High = 5.42
Testosterone LAA/ Male Hill Slope	0.85	0.394	Low = 2.18 High = 2.44
Testosterone RAA/ Male Top Force	4.39	**0.000**	Low = 0.97 High = 0.79
Testosterone RAA/ Male pCa 50	1.24	0.217	Low = 5.48 High = 5.43
Testosterone RAA/ Male Hill Slope	0.81	0.416	Low = 1.74 High = 1.96
Testosterone LAA/ Female Top Force	0.24	0.807	Low = 1.06 High = 1.06
Testosterone LAA/ Female pCa 50	0.10	0.918	Low = 5.41 High = 5.42
Testosterone LAA/ Female Hill Slope	0.33	0.745	Low = 2.12 High = 2.33
Testosterone RAA/ Female Top Force	3.34	**0.001**	Low = 0.93 High = 0.80
Testosterone RAA/ Female pCa	0.78	0.435	Low = 5.48 High = 5.43
Testosterone RAA/ Female Hill Slope	0.20	0.842	Low = 1.72 High = 1.96
E/T ratio LAA/ Male Top Force	0.26	0.795	Low = 1.06 High = 1.05
E/T ratio LAA/ Male pCa 50	0.36	0.719	Low = 5.41 High = 5.42
E/T ratio LAA/ Male Hill Slope	0.04	0.970	Low = 2.33 High = 2.24
E/T ratio RAA/ Male Top Force	2.06	**0.04**	Low = 0.82 High = 0.94
E/T ratio RAA/ Male pCa 50	0.20	0.842	Low = 5.46 High = 5.45
E/T ratio RAA/ Male Hill Slope	0.14	0.886	Low = 1.80 High = 1.85
E/T ratio LAA/ Female Top Force	4.71	**0.000**	Low = 1.03 High = 1.11
E/T ratio LAA/ Female pCa 50	1.23	0.219	Low = 5.51 High = 5.35
E/T ratio LAA/ Female Hill Slope	0.06	0.949	Low = 1.61 High = 2.27
E/T ratio RAA/ Female Top Force	2.17	**0.030**	Low = 0.98 High = 0.59
E/T ratio RAA/ Female pCa 50	1.18	0.239	Low = 5.37 High = 5.57
E/T ratio RAA/ Female Hill Slope	0.18	0.856	Low = 1.66 High = 1.86

Low estradiol values were associated with borderline higher force values in right-sided myofilaments of men (p = 0.056) but not in left-sided myofilaments (p = 0.1). In women low estradiol was associated with higher top force values in both right- and left-sided myofilaments.

Low testosterone levels correlated with higher top force values in both men’s and women’s right-sided myofilaments only.

Higher top force values were noted in right-sided myofilaments of male patients with higher E/T ratios (p 0.04). In women, higher E/T ratio was associated with higher top force values in left-sided myofilaments (p 0.000) but lower top force values in right-sided myofilaments.

## Discussion

To the best of our knowledge, this is the first study to describe the impact of 17ß-estradiol, testosterone serum concentration and their ratio on the contractile function of left and right auricular myofilaments in patients scheduled for aortocoronary bypass grafting.

We found that low estradiol serum levels were associated with higher force values in right-sided myofilaments in men and women. Since diabetes mellitus, AF and POD had a negative effect on force development of both right and left auricular myofilaments these patients’ samples were excluded from further testing. The negative inotropic effect of these comorbid factors has been described before [13–16].

After exclusion of samples from such patients low 17ß-estradiol was associated with higher left auricular top force values in women, but not in men. In contrast, right auricular top force values revealed higher values with low 17ß-estradiol in both genders. Low testosterone was associated with higher right auricular myofilament top force values in both men and women. There was no effect of testosterone on left-sided myofilament force values. In men higher E/T ratio was associated with higher top forces in right auricular myofilaments only. For women higher E/T ratio was associated with higher top force values in left-sided but lower top force values in right-sided myofilaments.

Our results correspond with previous studies. Sitzler et al. observed a negative inotropic effect on force development after exposure to 17ß Estradiol in human right atrial tissue samples [8]. In his study, addition of testosterone on the specimen had no effect on contractility leading to the assumption of a calcium antagonistic effect, possibly due to interaction with 1,4 dihydropyridine binding site of L-type calcium channels, of 17ß estradiol on right atrial myofilaments [8, 17, 18]. Jiang et al. showed an inhibition of the slow calcium inward current induced by 17ß-estradiol in isolated guinea-pig ventricular myocytes [19], This inhibition was not present after exposure to testosterone, supporting a negative inotropic effect of 17ß-estradiol [8, 17–19]. This effect might contribute to the reduced force values associated with higher estradiol concentrations in our study group. But furthermore estradiol also seems to have a direct effect on the contractile proteins, since estradiol seems to attenuate atrial essential myosin light chain expression in cardiomyocytes exerting a negative inotropic effect [20].

Ventetuolo et al. observed genetic variations in estradiol metabolism associated with right auricular morphology. This supports our results that an effect of 17ß estradiol was present on right atrial myofilaments only. The observed effect of higher testosterone concentrations leading to low top force values in right atrial myofilaments from both sexes can be due to an inflammatory effect of testosterone with subsequent cardiac remodeling and reduced RV function [3, 21, 22]. Furthermore, the presence of different modifications of androgen receptor genotypes in men associated with reduced RVEF may support our results [10]. We did not find any association of lower testosterone and force values in left auricular myofilaments in either gender. Testosterone may influence L-type calcium channels [23, 24]. However contradictory observation s about the effect of testosterone on L-type calcium channels have been published [23, 24]. Golden et al. reported an increase of mRNA levels of L-type calcium channels after testosterone application, which might indicate increased contractility [23]. In contrast Gupte et al. showed that testosterone metabolites blocks L-type calcium channels in isolated rat hearts with Langendorff perfusion and inhibits cardiac contractility [25]. Moreover, testosterone acts not only via androgen receptors but also via genomic pathways [26].

Thus conflicting results clearly demonstrate that the influence of testosterone on cardiac contractility is not fully understood and that further research is mandatory.

Since sex hormones interact, their balance could be more relevant than single hormone serum concentration and assessment of E/T ratio on myocardial performance a valuable process [6]. In our study population higher E/T ratio was associated with higher right auricular top force values in men but had no effect on left auricular myofilaments. In contrast, a higher E/T ratio in women correlated with reduced right auricular top force values and higher left auricular top force values indicating a gender and side (right atrial or left atrial) specific effect. The effect of E/T ratio in men is supported by Ventetuolo et al. They showed that higher E/T ratio was associated with lower RV volume in men, implicating better RV function [9]. Since human studies concerning sex hormones and their impact on myocardial contractility in women are lacking, one has to rely on animal studies up to now.

However, these results are even more conflicting. A positive effect of 17ß estradiol on cardiac contractility was reported with different animal models [27–31]. In all models ovariectomy had no effect on cardiac function. Subsequent estrogen replacement had either no effect or resulted in reduced cardiac function [27–31]. One has to consider if results from animal models might be species specific, since myosin isoenzymes and contractility are species-specific. Therefore, these results may only be partially applicable to humans [32].

### Limitations

Several limitations must be noted. First, despite being one of the largest studies with human tissue, the sample size is still too small to draw firm conclusions concerning the influence of sex hormones on left and right auricular myofilaments. This limits the statistical power of the results concerning E/T ratio in the female group. Furthermore the female sample size is very small and the advanced age combined with the postmenopausal hormone status is not representative for all females, which might have influenced the results. Second, a possible impact of hormone replacement therapy or hysterectomy/ ovariectomy on cardiac function and hormone status could be of high interest, however none of our patients received hormone replacement therapy or underwent hysterectomy or ovariectomy. We cannot exclude that medication like ACE inhibitors, which decrease pre- and afterload, might have influenced the contractile behavior of the skinned fibers through decreased wall stress. Third, we used human atrial tissue from left and right auricle. If these results can be extrapolated to ventricular tissue still is a matter of discussion although Vannier et al. observed similar contractile properties of human atrial and ventricular tissue thereby concluding a transferability of these results to ventricular tissue [33]. Fourth, all female patients in our study were postmenopausal, so 17ß-estradiol serum values were decreased and analyzed with a mean cut off. A control group of premenopausal women would be desirable to assess differences of 17ß-estradiol on contractility. Furthermore, the total number of women was low in our study, reflecting a well-known great dilemma of studies. Fifth, previous myocardial infarction as well as reduced left ventricular function in fermales might have also influenced the contractile properties of the myofilaments. Sixth, operator-specific treatment of the tissue samples with possible damage to the trabeculae cannot be excluded, although harvesting protocols were designed with a high degree of standardization. Seventh, we cannot estimate the influence of pathologic conditions like coronary heart disease, a chronic disease associated with inflammation, as an impact factor on cardiac properties of the myofilaments in opposite to normal physiological conditions. We have to admit restrictively that the observed correlations of sex hormones and cardiac function on level of the myofilaments might differ in healthy persons without cardiac disease.

Despite these limitations our study may serve as catalyst for future mechanistic and observational studies to define the influence of sex hormones on right and left heart contractility.

## Conclusions

In summary, patients’ comorbidities influence left and right sided contractility and may disguise or eliminate the effect of sex hormones on cardiac contractility. A sex hormone dependent influence is evident with different effects on the left and right ventricle. The E/T ratio and its impact on myofilament top force values showed divergent results for men and women and may partially explain gender differences in patients with cardiovascular disease.

Our preliminary results should trigger further studies on the impact of sex hormones on cardiac contractility in a gender-dependent manner.

## Data Availability

The datasets used and analyzed during the current study are available from the corresponding author on reasonable request.

## Abbreviations

ADMA: Asymmetric Dimethylarginine
AF: Atrial fibrillation
BMI: Body mass index
CABG: Coronary artery bypass grafting
CAD: Coronary Artery Disease
CRP: C-reactive protein
DM: Diabetes mellitus
E/T ratio: 17ßEstradiol/Testosterone ratio
GFR: Glomerular filtration rate
HbA1C: Glycated haemoglobin
LA: Left atrium
LVEF: Left ventricular ejection fraction
NT-ProBNP: N-terminal pro brain natriuretric peptid
POD: Peripheral occlusive disease
RA: Right atrium
RAA: Right atrial appendage
sPAP: Systolic pulmonary artery pressure
SR: Sinus rhythm
TAPSE: Tricuspid annular plane systolic excursion

## References

[1] Parks RJ, Howlett SE (2013). Sex differences in mechanisms of cardiac excitation-contraction coupling. Pflugers Arch.

[2] Schwertz DW, Beck JM, Kowalski JM, Ross JD (2004). Sex differences in the response of rat heart ventricle to calcium. Biol Res Nurs.

[3] Bening C, Hamouda K, Leyh RBMC (2016). Sex differences in volume overload in skinned fibers. Cardiovasc Disord.

[4] Bening C, Weiler H, Vahl CF (2013). Effects of gender, ejection fraction and weight on cardiac force development in patients undergoing cardiac Surgery–an experimental examination. J Cardiothorac Surg.

[5] Ventura-Clapier R, Dworatzek E, Seeland U, Kararigas G, Arnal JF, Brunelleschi S (2017). Sex in basic research: concepts in the cardiovascular field. Cardiovasc Res.

[6] Dai W, Li Y, Zheng H (2012). Estradiol/Testosterone imbalance: impact on coronary Heart Disease risk factors in postmenopausal women. Cardiology.

[7] Witayavanitkul N, Woranush W, Bupha-Intr T, Wattanapermpool J (2013). Testosterone regulates cardiac contractile activation by modulating SERCA but not NCX activity. Am J Physiol Heart Circ Physiol.

[8] Sitzler G, Lenz O, Kilter H, La Rosee K, Böhm M (1996). Investigation of the negative inotropic effects of 17 beta-oestradiol in human isolated myocardial tissues. Br J Pharmacol.

[9] Ventetuolo CE, Ouyang P, Bluemke DA, Tandri H, Barr RG, Bagiella E (2011). Sex hormones are associated with right ventricular structure and function: the MESA-right ventricle study. Am J Respir Crit Care Med.

[10] Ventetuolo CE, Mitra N, Wan F, Manichaikul A, Barr RG, Johnson C (2016). Oestradiol metabolism and androgen receptor genotypes are associated with right ventricular function. Eur Respir J.

[11] Subramanya V, Zhao D, Ouyang P, Lima JA, Vaidya D, Ndumele CE (2018). Sex hormone levels and change in left ventricular structure among men and post-menopausal women: the multi-ethnic study of Atherosclerosis (MESA). Maturitas.

[12] Hill AV (1910). The possible effects of the aggregation of the molecules of haemoglobin on its dissociation curves. J Pyhsiology.

[13] Belus A, Piroddi N, Ferrantini C, Tesi C, Cazorla O, Toniolo L (2010). Effects of chronic atrial fibrillation on active and passive force generation in human atrial myofibrils. Circ Res.

[14] Eiras S, Narolska NA, van Loon RB, Boontje NM, Zaremba R, Jimenez CR (2006). Alterations in contractile protein composition and function in human atrial dilatation and atrial fibrillation. J Mol Cell Cardiol.

[15] Jweied EE, McKinney RD, Walker LA, Brodsky I, Geha AS, Massad MG (2005). Depressed cardiac myofilament function in human Diabetes Mellitus. Am J Physiol Heart Circ Physiol.

[16] Kim W, Kang TS (2018). Effect of successful revascularization on left ventricular diastolic dysfunction in patients with aortoiliac occlusive Disease. Med (Baltim).

[17] Brown AM, Kunze DL, Yatani A (1984). The agonist effect of dihydropyridines on ca channels. Nature.

[18] Collins P, Rosano GM, Jiang C, Lindsay D, Sarrel PM, Poole-Wilson PA (1993). Cardiovascular protection by oestrogen–a calcium antagonist effect?. Lancet.

[19] Jiang C, Poole-Wilson PA, Sarrel PM, Mochizuki S, Collins P, MacLeod KT (1992). Effect of 17 beta-oestradiol on contraction, Ca2 + current and intracellular free Ca2 + in guinea-pig isolated cardiac myocytes. Br J Pharmacol.

[20] Duft K, Schanz M, Pham H, Abdelwahab A, Schriever C, Kararigas G (2016). 17β-Estradiol-induced interaction of estrogen receptor α and human atrial essential myosin light chain modulates cardiac contractile function. Basic Res Cardiol.

[21] Nahrendorf M, Frantz S, Hu K, von zur Muhlen C, Tomaszewski M, Scheuermann H (2003). Effect of testosterone on post-myocardial infarction remodeling and function. Cardiovasc Res.

[22] Kłapcińska B, Jagsz S, Sadowska-Krępa E, Górski J, Kempa K, Langfort L (2008). Effects of castration and testosterone replacement on the antioxidant defense system in rat left ventricle. J Physiol Sci.

[23] Golden KL, Marsh JD, Jiang Y (2004). Testosterone regulates mRNA levels of calcium regulatory proteins in cardiac myocytes. Horm Metab Res.

[24] Scragg JL, Jones RD, Channer KS, Jones TH, Peers C (2004). Testosterone is a potent inhibitor of L-type ca(2+) channels. Biochem Biophys Res Commun.

[25] Gupte SA, Tateyama M, Okada T, Oka M, Ochi R, Epiandrosterone (2002). A metabolite of Testosterone Precursor, blocks L-type calcium channels of ventricular myocytes and inhibits myocardial contractility. J Mol Cell Cardiol.

[26] Er F, Michels G, Brandt MC, Khan I, Haase H, Eicks M, Lindner M, Hoppe UC (2007). Impact of testosterone on cardiac L-type calcium channels and Ca2 + sparks: acute actions antagonize chronic effects. Cell Calcium.

[27] Alecrin IN, Aldrighi JM, Caldas MA, Gebara OC, Lopes NH, Ramires JA (2004). Acute and chronic effects of oestradiol on left ventricular diastolic function in hypertensive postmenopausal women with left ventricular diastolic dysfunction. Heart.

[28] Fenkci V, Yilmazer M, Alpaslan M, Onrat E, Fenkci S (2003). The short-term effects of different regimens of hormone replacement therapy on left ventricular structure and performance in healthy postmenopausal women. A prospective, controlled echocardiographic study. Gynecol Obstet Invest.

[29] Schaible TF, Malhotra A, Ciambrone G, Scheuer J (1984). The effects of gonadectomy on left ventricular function and cardiac contractile proteins in male and female rats. Circ Res.

[30] Bowling N, Bloomquist WE, Cohen ML, Bryant HU, Cole HW, Magee DE (1997). Effects of prolonged ethinyl estradiol treatment on calcium channel binding and in vivo calcium-mediated hemodynamic responses in ovariectomized rats. J Pharmacol Exp Ther.

[31] Patterson E, Ma L, Szabo B, Robinson CP, Thadani U (1998). Ovariectomy and estrogen-induced alterations in myocardial contractility in female rabbits: role of the L-type calcium channel. J Pharmacol Exp Ther.

[32] Morano I, Arndt H, Gärtner C, Rüegg JC (1988). Skinned fibers of human atrium and ventricle: myosin isoenzymes and contractility. Circ Res.

[33] Vannier C, Veksler V, Mekhfi H, Mateo P, Ventura-Clapier R (1996). Functional tissue and developmental specificities of myofibrils and mitochondria in cardiac muscle. Can J Physiol Pharmacol.

